# Supplementary material for: Lipid desaturation regulates the balance between self-renewal and differentiation in mouse blastocyst-derived stem cells
Source: Cell Death Dis. 2022 Dec 7;13(12):1027. doi: 10.1038/s41419-022-05263-0 (PMC9729213; doi:10.1038/s41419-022-05263-0)
Supplement: Supplementary file 2 — Supplemental Material [file 41419_2022_5263_MOESM2_ESM.pdf]

Lipid Desaturation Regulates the Balance between Self-renewal and Differentiation in Mouse Blastocyst-derived Stem Cells

Chanchal Thomas Mannully[1], Reut Bruck-Haimson[1], Anish Zacharia[1], Paul Orih[1], Alaa Shehadeh[1], Daniel Saidemberg[1], Natalya M Kogan[1], Sivan Alfandary[1], Raphael Serruya[1], Arie Dagan[1], Isabelle Petit[2]; Arieh Moussaieff[1]*

1. The Institute for Drug Research, The Hebrew University of Jerusalem, Jerusalem 9112102, Israel.
2. Sorbonne Université, Inserm, Centre de Recherche Saint-Antoine, CRSA, F-75012 Paris, France.

Supplementary Information

The following file contains supplementary material for the paper "**Lipid Desaturation Regulates the Balance between Self-renewal and Differentiation in Mouse Blastocyst-derived Stem Cells**".

This file is composed of:

- Supplementary figures and relative supplementary figure legends (6 figures)
- Uncropped Western blot pictures
- Supplementary tables (3 tables)

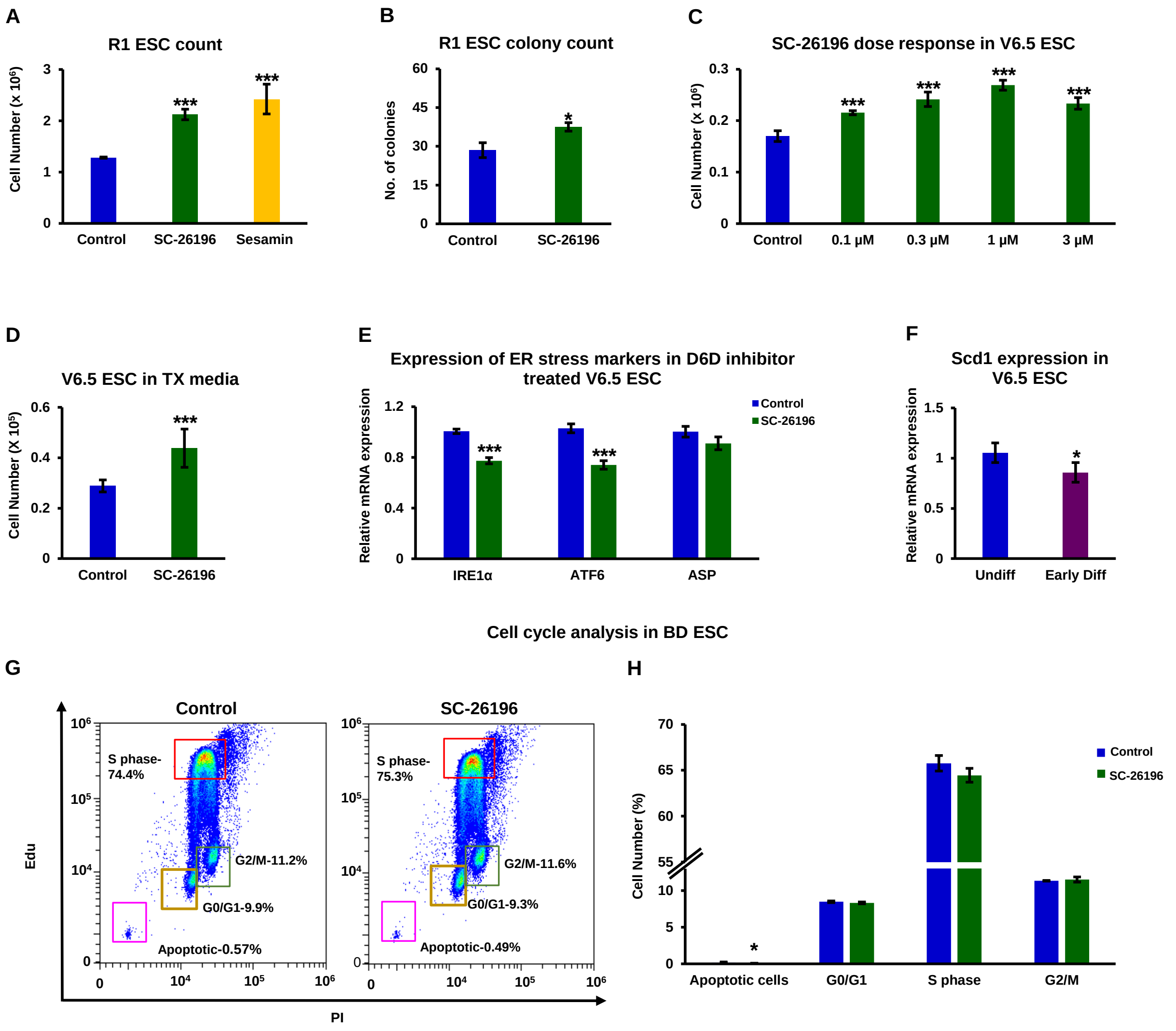

Figure S1. ER stress-related apoptosis in ESCs due to polyunsaturation of lipids is not cell line specific, and not dependent on cell cycle. To confirm the non-cell-line-specific nature of our results, we performed experiments with further ESC lines (R1 and V6.5; A-F). We also studied the effect of D6D inhibition on the ESC cell cycle (using the HA cell line used for the experiments shown in the main Figures), as an optional explanation for the increased proliferation rate. ESCs were treated with the specific D6D inhibitor SC-26196 (0.2 μM) or the specific D5D inhibitor sesamin (10 μM), or DMSO vehicle, for 48 hour in feeder free culture. Cell counting was carried out using CytoSmart cell counter and validated by manual counting (A; n = 4). Colony numbers per field were estimated under the microscope (B; 5 fields taken for each well, for 4 wells). Dose response evaluation of the influence of D6D inhibition on cell proliferation rate was performed with 0.1-3 μM SC-26196 (C; n = 4). The viability of ESCs was also assessed in Tx medium, as in A

(D; n = 4). The expression of ER stress markers was measured by RT-qPCR (E; n = 4). *Scd1* expression in early differentiating V6.5 ESCs was measured by RT-qPCR (F; n = 4). Cell cycle was analyzed in HA ESCs following inhibition of D6D by SC-26196 for 24 hours (G, H; n = 4). Data are presented as mean ± SEM. *, $P < 0.05$; **, $P < 0.01$; ***, $P < 0.001$.

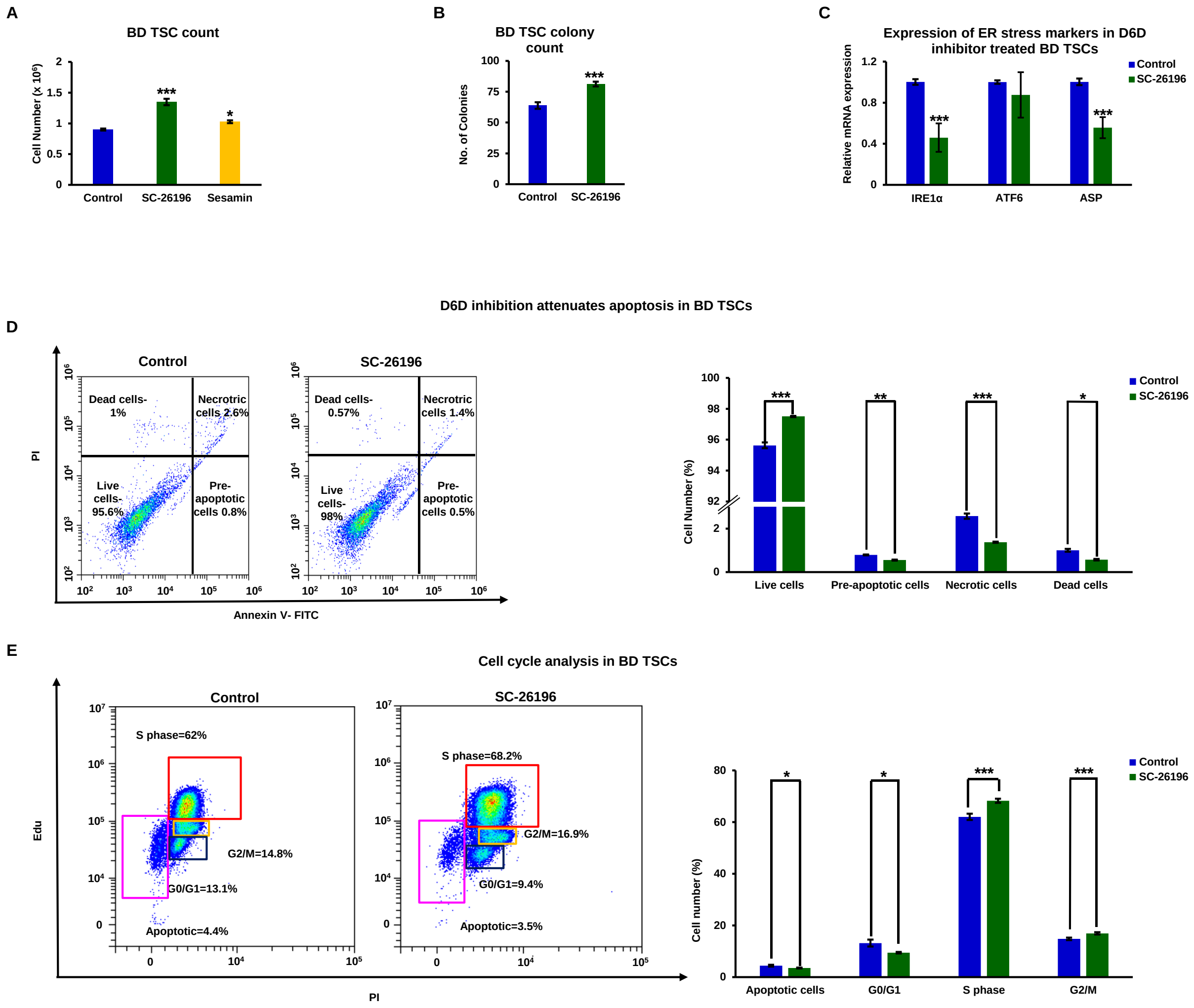

Figure S2. ER stress-related apoptosis and changes in cell cycle rate in TSCs due to polyunsaturation of lipids are not cell line specific. To confirm the non-cell-line-specific nature of our results, we performed experiments with a second blastocyst derived TSC line (Homo BL6 background). TSCs were treated with the specific D6D inhibitor SC-26196 (0.2 µM) or the specific D5D inhibitor sesamin (10 µM) (A-E) or DMSO vehicle for 48 hours in feeder free culture. Cell counting was carried out using CytoSmart cell counter, and validated by manual counting (A). Colony numbers per field were estimated under the microscope (B; 5 fields taken for each well, for 4 wells). The expression of *IRE1*, *ATF6* and *ASP* was examined by RT-qPCR following D6D inhibition for 24 hours (C). Apoptosis was assessed in TSCs following D6D inhibition for 24 hours using Annexin V/PI staining (D). The numbers in each quadrant represent the percentage of cells. Cell cycle was analyzed following inhibition of D6D by SC-26196 for 24 hours (E). n = 4; Data are presented as mean ± SEM. *, $P < 0.05$; **, $P < 0.01$; ***, $P < 0.001$.

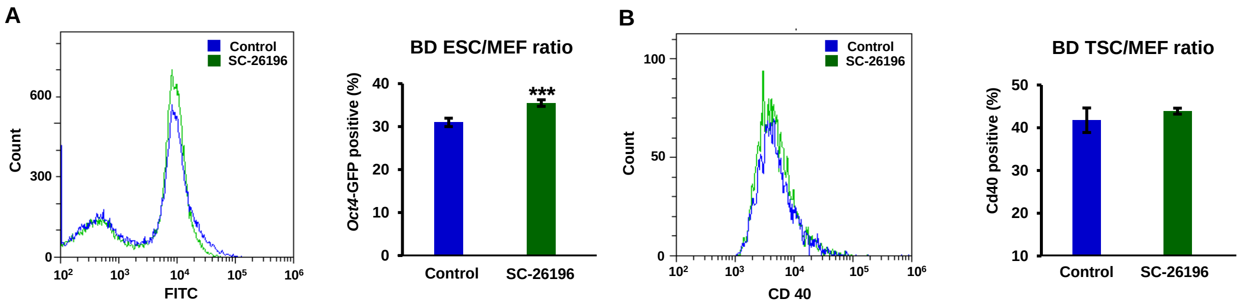

Figure S3: Influence of D6D on SC proliferation rate in feeder cell-based cultures. FACS analyses were carried out to assess the proliferation of ESCs and TSCs in co-culture with MEF feeder cells. The proliferation rate of ESCs and TSCs treated with SC-26196 (A-B; 0.2 µM) for 48 hours, was compared to their DMSO control as a ratio of SC to MEF number in culture. ESCs proliferation was assessed as a ratio of *Oct4*-GFP labeled cells to non-labeled cells (A); TSCs proliferation was assessed as a ratio of CD40-labeled cells to non-labeled cells (B). n = 4. Data are presented as mean ± SEM. *, $P < 0.05$; **, $P < 0.01$; ***, $P < 0.001$.

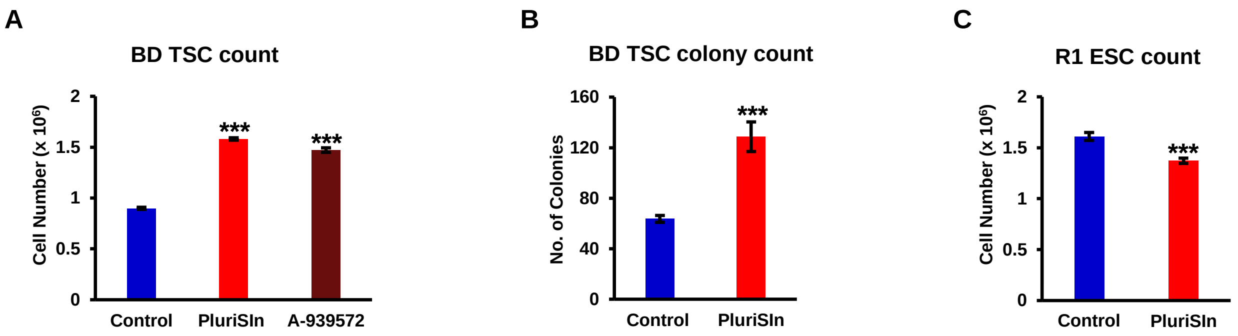

Figure S4: The increase in viability of TSCs, whereas decrease of ESC viability by Scd1 inhibition is not cell line specific. Following treatment with Scd1 specific inhibitor (PluriSIn 1; 20 µM), or DMSO vehicle, for 48 hours in feeder free culture, cell counting was carried out using CytoSmart cell counter, and further validated by manual counting of TSCs (Homo BL6 background; A) and ESCs (R1; C). Colony numbers per field were estimated under the microscope (B; 5 fields taken for each well, for 4 wells). n = 4. Data are presented as mean ± SEM. *, $P < 0.05$; **, $P < 0.01$; ***, $P < 0.001$.

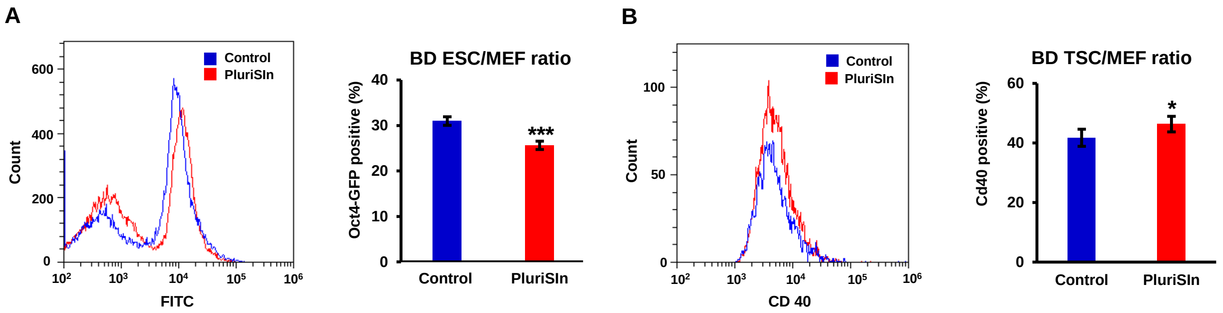

Figure S5: The influence of Scd1 on SC proliferation rate in feeder cell-based culture. FACS analysis was used to quantify the proliferation of ESCs and TSCs in co-culture with MEF feeder cells. The proliferation rate of ESCs (A) and TSCs (B) treated with PluriSIn 1 (20 µM) for 48 hours, was compared to their DMSO control as a ratio to MEFs in culture. ESCs proliferation was assessed as a ratio of *Oct4*-GFP labeled cells to non-labeled cells; TSCs proliferation was assessed as a ratio of CD40-labeled cells to non-labeled cells. n = 4. Data are presented as mean ± SEM. *, $P < 0.05$; **, $P < 0.01$; ***, $P < 0.001$.

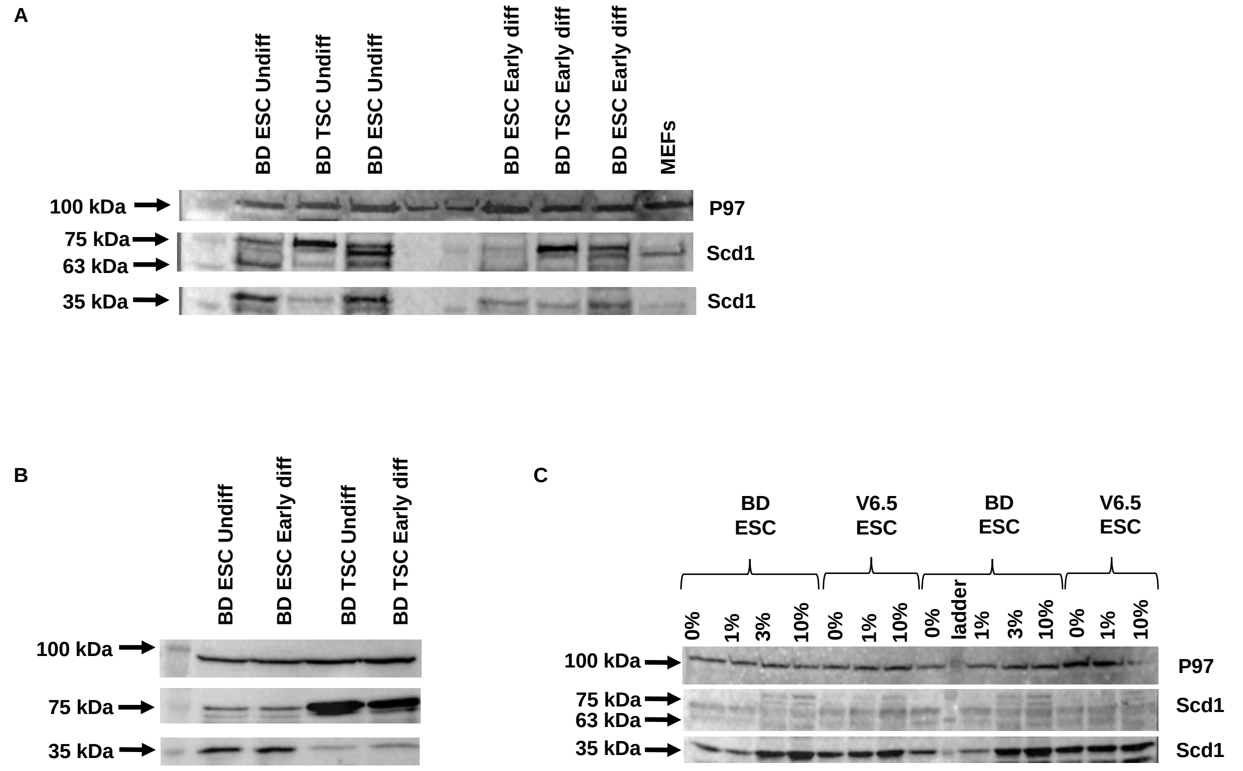

Figure S6: Scd1 protein is expressed as a 37 kDa variant in ESCs, whereas largely expressed as a 70 kDa in TSCs. BD ESCs and TSCs were incubated in ESC medium or Tx medium, respectively (A), or both in Tx medium (B), and the expression of Scd1 was assessed by Western blot analysis. BD ESCs and V6.5 ESCs were incubated in medium containing 0-10% fetal bovine serum for 48 hours. Scd1 protein expression was assessed by Western blotting (C).

Original uncropped western blot images

Uncropped western blot for the supplementary figure S6-A

Scd1

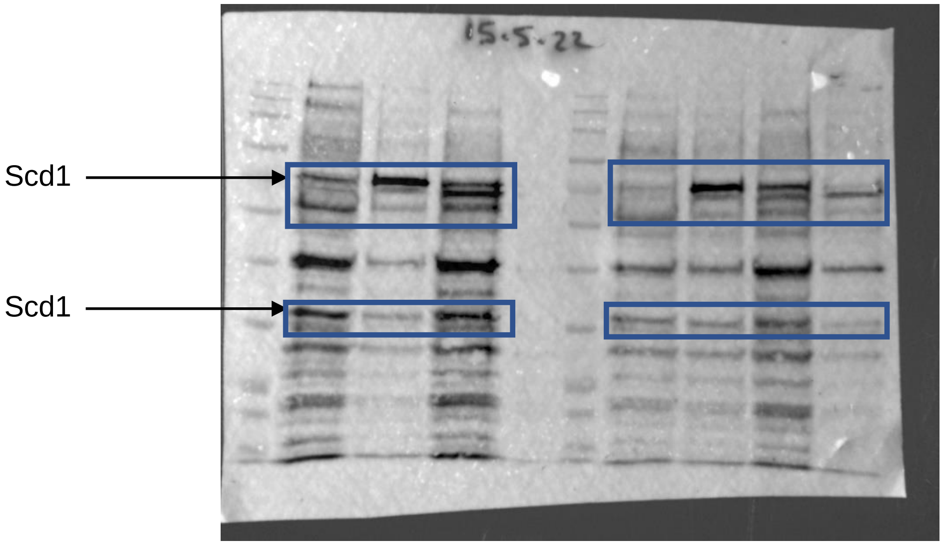

P97 (Loading control)

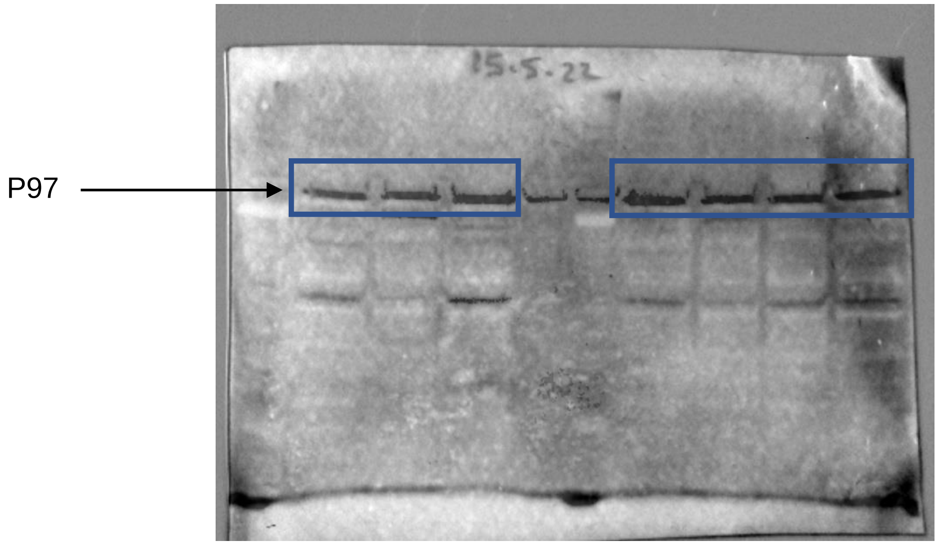

Uncropped western blot for the Supplementary Figure S6-B

Scd1

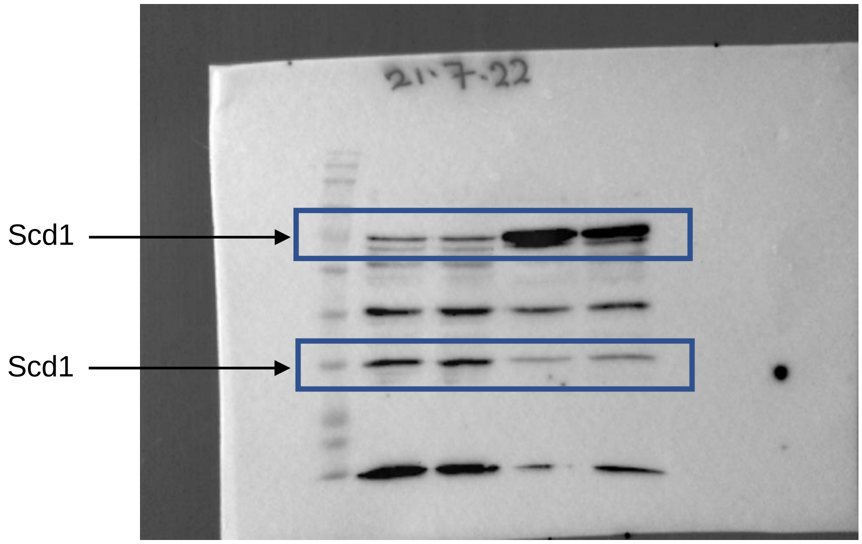

P97 (Loading control)

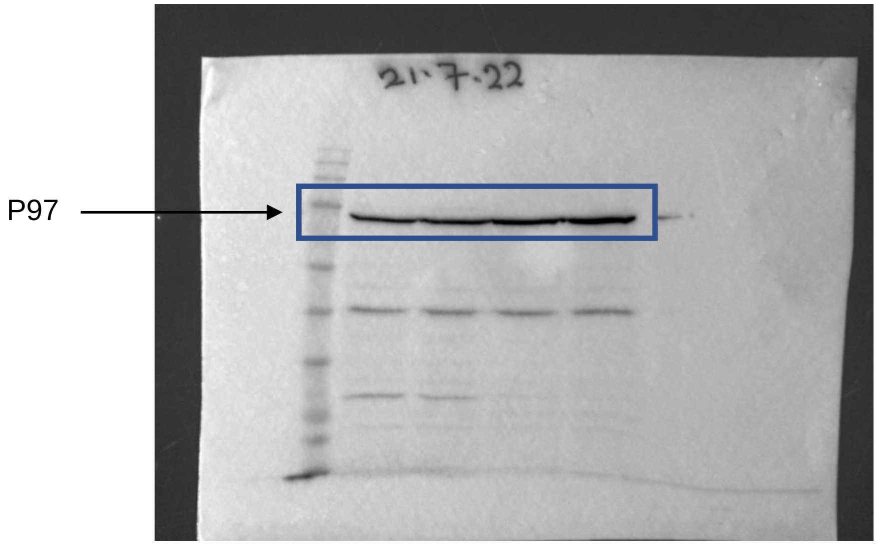

Uncropped western blot for the Supplementary Figure S6-C

Scd1

Scd1

Scd1

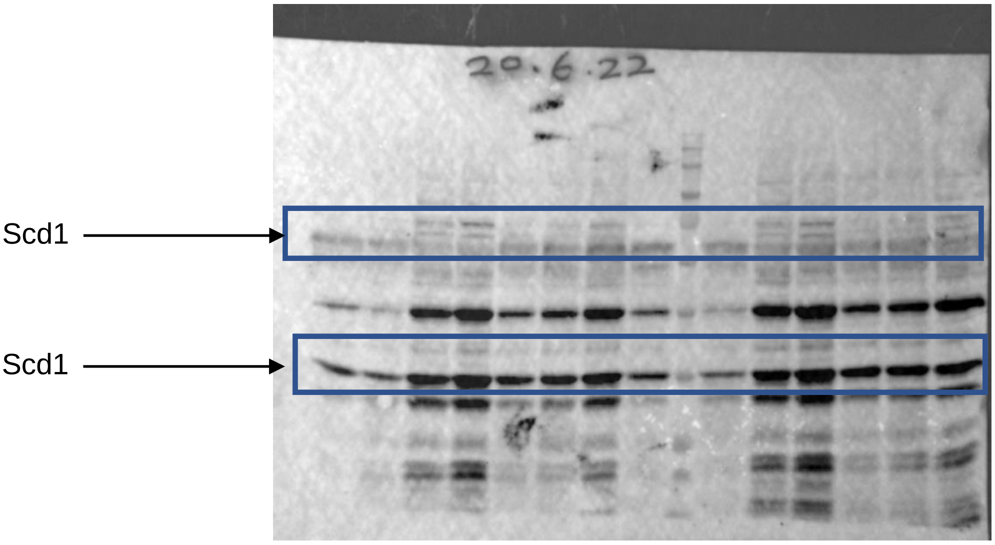

P97 (Loading control)

P97

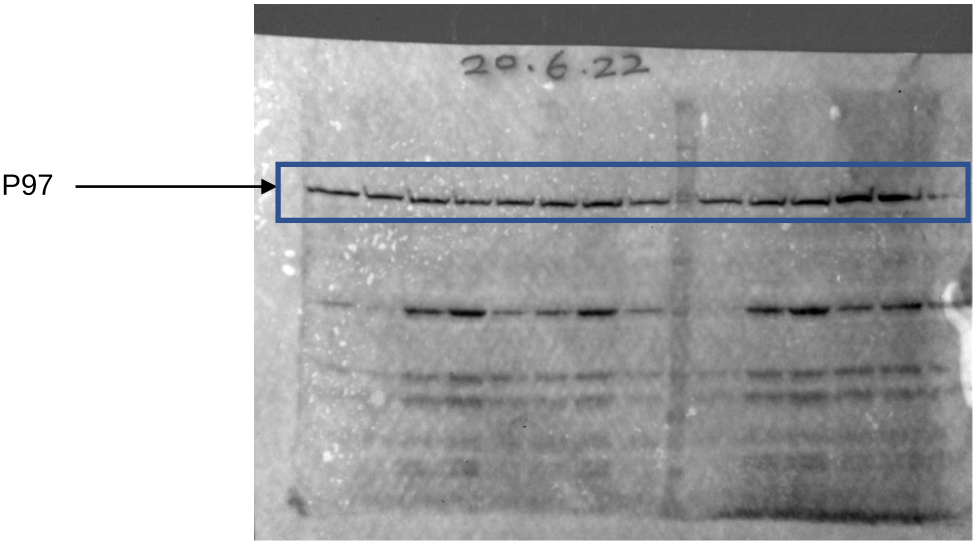

Table S1: Discriminant lipid species identified in the early differentiated ESCs and TSCs

Lipid Species	Identification	Empirical Formula	m/z
1	PE(22:5/P-16:0)	$C_{43}H_{76}NO_7P$	748.52 (M-H)
2	PC(16:0/20:3)	$C_{43}H_{80}NO_8P$	828.57 (M+FA-H)
3	PE(P-16:0/18:4)	$C_{39}H_{70}NO_8P$	694.48 (M-H)
4	PI(20:4/18:0)	$C_{47}H_{83}O_{13}P$	885.54 (M-H)
5	PE(20:3/P-18:0)	$C_{45}H_{82}NO_7P$	838.59 (M+FA-H)
6	PI(20:3/18:0)	$C_{47}H_{85}O_{13}P$	887.56 (M-H)
7	PE(18:0/20:4)	$C_{43}H_{78}NO_8P$	766.53 (M-H)
8	PE(20:4/P-18:0)	$C_{43}H_{78}NO_7P$	750.52 (M-H)
9	PE(22:4/P-18:0)	$C_{43}H_{80}NO_7P$	776.56 (M-H)
10	PC(16:0/20:4)	$C_{44}H_{80}NO_8P$	826.55 (M+FA-H)
11	PE(22:6/18:0)	$C_{45}H_{78}NO_8P$	790.53 (M-H)
12	PE(16:0/22:6)	$C_{43}H_{74}NO_8P$	762.50 (M-H)
13	PE(20:5/18:1)	$C_{43}H_{74}NO_8P$	762.50 (M-H)
14	PE(22:6/P-16:0)	$C_{43}H_{74}NO_8P$	746.51 (M-H)
15	PG (20:4/18:3)	$C_{44}H_{73}O_{10}P$	837.49 (M+FA-H)
16	PE(22:6/16:1)	$C_{43}H_{72}NO_8P$	760.49 (M-H)
17	PG(18:1/22:5)	$C_{46}H_{79}O_{10}P$	821.54 (M-H)
18	PE(P-18:0/22:6)	$C_{45}H_{78}NO_7P$	774.54 (M-H)
19	PE(16:1/20:3)	$C_{41}H_{74}NO_8P$	738.50 (M-H)
20	PI (18:1/18:1)	$C_{45}H_{83}O_{13}P$	861.54 (M-H)
21	PG (16:1/18:1)	$C_{40}H_{75}O_{10}P$	745.50 (M-H)
22	PC(14:0/18:1)	$C_{40}H_{78}NO_8P$	1508.09 (2M+FA-H)
23	PE(18:1/18:1)	$C_{23}H_{46}NO_7P$	742.53 (M-H)
24	SM(18:1/16:0)	$C_{39}H_{79}N_2O_6P$	747.56 (M+FA-H)
25	PG(18:0/18:1)	$C_{42}H_{81}O_{10}P$	775.54 (M-H)
26	PE(P-18:1/18:1)	$C_{41}H_{78}NO_7P$	726.54 (M-H)
27	PE(18:1/16:0)	$C_{39}H_{76}NO_8P$	716.52 (M-H)
28	PE(18:1/16:1)	$C_{39}H_{74}NO_8P$	715.51 (M-H)
29	PE(18:1/20:1)	$C_{43}H_{82}NO_8P$	770.56 (M-H)
30	PC(14:0/16:1)	$C_{38}H_{74}NO_8P$	748.54 (M+FA-H)
31	PG(18:1/18:1)	$C_{42}H_{78}O_{10}P$	773.53 (M-H)
32	Phospholipid (34:2)*	$C_{39}H_{74}NO_8P$	715.50 (M-H)

33	Phospholipid (34:1)*	$C_{41}H_{80}NO_8P$	744.54 (M-H)
34	Phospholipid (34:1)*	$C_{39}H_{76}NO_8P$	716.52 (M-H)
35	Phospholipid (36:3)*	$C_{42}H_{77}O_{10}P$	771.51 (M-H)
36	Phospholipid (40:6)*	$C_{46}H_{80}NO_8P$	850.55 (M+FA-H)
37	Phospholipid (34:3)*	$C_{42}H_{78}NO_8P$	800.54 (M+FA-H)
38	Phospholipid (38:4)*	$C_{44}H_{79}O_{10}P$	797.53 (M-H)
39	Phospholipid (36:1)*	$C_{44}H_{86}NO_8P$	832.60 (M+FA-H)

PE, Phosphatidylethanolamine; PC, Phosphatidylcholine; PI, Phosphatidylinositol; PG, Phosphatidylglycerol; SM, Sphingomyelin. The lipids were identified by MS^E. * Identified as phospholipids based on diagnostic ions (no full assignment).

Table S2: Temperature gradient for Gas chromatography

Rate (°C/min)	Value (°C)	Hold Time (Min)	Run Time (Min)
	50	5	5
10	170	0	17
3.5	250	3	42

Table S3: List of primers used for RT-qPCR

Mouse Genes	Forward Primer (Sequence: 5' to 3')	Reverse Primer (Sequence: 5' to 3')
Ubc	CAGCCGTATATCTTCCCAGACT	CTCAGAGGGATGCCAGTAATCTA
Hprt	ATTCACCCCCACTGAGACTG	AGGGCATATCCAACAACAAACTT
Scd1	TTCTTGCGATACACTCTGGTGC	CGGGATTGAATGTTCTTGTCGT
D6D1	TCATCGGACACTATTCGGGAG	GGGCCAGCTCACCAATCAG
D6D2	GACCGTGGCAAAAGCTCTCA	CTGTGACGAGGGTAGGAATCC
D5D	ACCCAGCTTTGAACCCACC	GAGGCCCATTCGCTCTACTG
Nanog	AAACCAGTGGTTGAAGACTAGCAA	GGTGCTGAGCCCTTCTGAATC
Oct4	GCTTGGGCTAGAGAAGGATGTG	TGGCGCCGGTTACAGAAC
Sox2	GCGGCGGAAAACCAAGA	CCGGGAAGCGTGTACTTATCC
Cdx2	CACTTTAGTCGATACATCACCATC	GATTTTCCTCTCCTTGGCTCT
Elf5	GGACCGATCTGTTCAGCAAT	GGGTGCACTGATGTCCAGTA
Tfap2c	CAGGTCACTCTCCTCACGTC	CAGCTTCGCAGACATAGGC
Eomes	CGGCAAAGCGGACAATAACA	GGAGCCAGTGTTAGGAGATTC
Asp	CTAGCCATCACTTATGGTTTGGT	TGCCCAGATTACTAAACTGCCA
Atf3	CCCCTGGAGATGTCAGTCAC	CGGTGCAGGTTGAGCATGT
Atf6	CAGTTGCTCCATCTCCTCTCC	TGGGACACTGGCATTGGTTTG
Ire1α	GAGCAAGCTAACGCCTACTCTGT	CACCATTGAGGGAGAGGCATA
